# Reevaluating the role of interferon‐beta in psoriasis pathogenesis: A registry‐based self‐controlled study

**DOI:** 10.1111/1346-8138.17338

**Published:** 2024-06-11

**Authors:** Ida M. Heerfordt, Elisabeth Framke, Josefine Windfeld‐Mathiasen, Mette Mogensen, Rasmus Huan Olsen, Melinda Magyari, Henrik Horwitz

**Affiliations:** ^1^ Department of Clinical Pharmacology Copenhagen University Hospital – Bispebjerg and Frederiksberg Copenhagen Denmark; ^2^ Danish Multiple Sclerosis Registry, Department of Neurology Copenhagen University Hospital – Rigshospitalet Glostrup Denmark; ^3^ Department of Clinical Medicine University of Copenhagen Copenhagen Denmark; ^4^ Department of Dermatology Copenhagen University Hospital – Bispebjerg and Frederiksberg Copenhagen Denmark

**Keywords:** immunomodulation, inflammatory diseases, interferon‐beta, psoriasis pathogenesis, skin

## Abstract

Interferon‐beta has been suggested as a trigger of psoriasis, yet a systematic investigation is lacking. This study aimed to assess the risk of developing psoriasis following interferon‐beta treatment, utilizing a pharmaco‐epidemiological approach to investigate the role of interferon‐beta in psoriasis pathogenesis. We included all treatment‐naïve patients with multiple sclerosis (MS) in Denmark who initiated interferon‐beta treatment for MS from January 1996 to June 2023. These patients were compared to a control cohort of patients with MS treated with other disease‐modifying drugs. We compared the incidence rates of psoriasis before and during the treatment. Data for this study were extracted from the Danish MS Registry and integrated with information from other national Danish health registries. Among 7174 patients treated with interferon‐beta, the incidence rate of psoriasis post‐treatment initiation was slightly higher (2.01 per 1000 person‐years) compared to the rate prior to treatment (1.67 per 1000 person‐years). This increase did not achieve statistical significance (*P* = 0.53), with an incidence rate ratio (IRR) of 1.20 (95% confidence interval [CI] 0.68–2.13). The control cohort showed an increase in psoriasis incidence post‐treatment initiation (3.12 per 1000 person‐years) compared to prior (1.11 per 1000 person‐years), with an IRR of 2.80 (95% CI 1.36–4.77, *P* = 0.0038). This registry‐based self‐controlled study does not support the theory that interferon‐beta acts as a trigger for psoriasis development.

## 1 INTRODUCTION

Psoriasis is a common chronic inflammatory skin disease with a multifactorial etiology.^1^ Although the exact cause of psoriasis is uncertain, it is known that both genetic and environmental factors play roles.^1^ Additionally, type 1 interferons, including interferon‐alpha and interferon‐beta, have been suggested as triggers of the disease.^2^ Type 1 interferons directly interact with cells resident in the skin, recruiting and influencing inflammatory cells.^2^ It is theorized that this interaction can trigger the inflammation associated with psoriasis.^2^ This theory is supported by several observations. First, case reports indicate that patients receiving recombinant interferon treatment for multiple sclerosis (MS) or viral infections have experienced worsening of existing psoriasis or new psoriasis development.^3^, ^4^ Furthermore, elevated levels of type I interferons have been detected in the blood and skin of patients with psoriasis.^5^, ^6^

MS is a severe and debilitating demyelinating disease, and 30 years ago interferon‐beta was introduced as a disease‐modifying drug (DMD) with moderate effectiveness for MS.^7^ Despite anecdotal evidence of interferon‐beta potentially inducing psoriasis in patients with MS,^3^, ^4^ a systematic investigation of this association is yet to be conducted.^2^ Thus, we aimed to assess psoriasis risk among a large cohort of patients with MS treated with interferon‐beta.

## 2 METHODS

### 2.1 Design

The study was a self‐controlled cohort study, assessing psoriasis incidence around interferon‐beta treatment initiation. We hypothesized that individuals starting interferon‐beta had similar psoriasis risk profiles, altered only by the treatment. The analysis focused on 2 years before and up to 2 years after treatment start. To account for other variables affecting psoriasis risk during this period, we compared these patients to others who began different MS DMDs, excluding those on dimethyl fumarate, methotrexate, azathioprine, and methylprednisolone because of their properties as psoriasis treatments.^8^

### 2.2 Data

Every resident in Denmark has a unique civil registration number (CPR) used for all healthcare system interactions.^9^, ^10^ The Danish MS Registry, dating back to 1956, includes data on all patients with MS in Denmark, such as sex, age, clinical details, and treatment information.^11^ Our study included treatment‐naïve patients initiating interferon‐beta or other DMDs, except for dimethyl fumarate, methotrexate, azathioprine, and methylprednisolone, from January 1, 1996, to June 25, 2023. DMDs that had been used for fewer than 50 individuals were excluded from the analysis.

We linked CPR numbers from the study participants with the Danish National Patient Register and the Danish National Prescription Registry.^9^, ^10^ The Danish National Patient Register documents psoriasis diagnoses from both hospital inpatient and outpatient visits, following the International Classification of Diseases, 10th Revision (ICD‐10) criteria.^10^ The Danish National Patient Register, however, does not capture data from general practitioners. The Danish National Prescription Registry is a comprehensive database that systematically records all prescription medications dispensed at pharmacies across Denmark classified under the Anatomical Therapeutic Chemical (ATC) system.^9^ For this study, we extracted data on prescriptions related to psoriasis treatment.

An event in the study was defined by either a primary diagnosis of psoriasis (ICD‐10 code L40) or a prescription of antipsoriatic treatment (ATC code D05A). Those meeting either criterion during the study were identified as cases. The study period spanned 2 years before and after interferon‐beta treatment initiation or discontinuation, excluding those with prior psoriasis, defined based on criterion one or two.

The exposure was the first DMD initiation for MS, with interferon‐beta as our primary interest. The control group included patients starting other DMDs detailed in Table 1.

**TABLE 1:** Distribution of treatment types among included patients.

Treatment	Individuals treated, *n*
Interferon‐beta	
Interferon‐beta‐1a	5975
Interferon‐beta‐1b	1140
Peginterferon‐beta‐1a	59
Other disease‐modifying drugs	
Fampridine	382
Fingolimod	169
Glatiramer acetate	787
Mitoxantrone	191
Natalizumab	484
Ocrelizumab	426
Ofatumumab	123
Rituximab	222
Teriflunomide	2152

### 2.3 Statistics

We employed a univariate Poisson regression model. In all analyses, a *P* value of less than 0.05, considered on a two‐sided basis, was deemed statistically significant, and all incidence rate ratios (IRRs) are reported alongside their 95% confidence intervals (CIs).

Additionally, to ensure the robustness of our analysis, a sensitivity analysis was conducted, limiting the observation time to a maximum of 1 year each for the periods before and during treatment.

Analyses were performed using SAS 9.4 (SAS Institute Inc).

### 2.4 Ethics

Danish law exempts non‐interventional register studies from ethics approval and patient consent. For privacy, results for groups under five individuals are not reported.

## 3 RESULTS

### 3.1 Interferon‐beta

We identified 7329 patients who initiated treatment with interferon‐beta. Of these, 155 had pre‐existing psoriasis, leaving 7174 for analysis. Characteristics of the cohort are available in Table 2.

**TABLE 2:** Cohort characteristics.

	Interferon‐beta	Other disease‐modifying drugs
Individuals, *n*	7174	4936
Female (%)	69.71	64.26
Mean age at treatment initiation, years (standard deviation)	38.25 (10.43)	41.10 (12.74)
Median year of treatment start (interquartile range)	2007 (2001–2010)	2017 (2014–2019)

In the 2‐year period leading up to interferon‐beta treatment, 24 patients were diagnosed with psoriasis during a total of 14 348 person‐years at risk, resulting in an incidence rate of 1.67 per 1000 person‐years. In the treatment period, 23 patients were diagnosed with psoriasis during a total of 11 428 person‐years at risk, leading to an incidence rate of 2.01 per 1000 person‐years. Thus, the incidence rate was numerically higher during treatment with interferon‐beta than in the period prior to treatment initiation but did not reach the threshold for statistical significance (*P* = 0.53). The IRR was 1.20 (95% CI 0.68–2.13).

### 3.2 Other DMDs

The control cohort consisted of 5088 treatment‐naïve patients who initiated with drugs other than interferon‐beta, dimethyl fumarate, methotrexate, azathioprine, and methylprednisolone against MS. Of these, 152 had pre‐existing psoriasis, leaving 4936 for analysis.

In the control cohort 11 patients were diagnosed with psoriasis in the 2‐year period leading up to treatment, resulting in an incidence rate of 1.11 per 1000 person‐years. In the treatment period 22 were diagnosed with psoriasis, leading to an incidence rate of 3.12 per 1000 person‐years, an incidence rate that is statistically different from before treatment (IRR 2.80, 95% CI 1.36–4.77). All results are displayed in Table 3.

**TABLE 3:** Psoriasis incidence rates before and during treatment.

Drug	Observation period	Incident psoriasis cases	Person‐years at risk	Psoriasis incidence rate, per 1000 person‐years	Incidence rate ratio	*P* value
Interferon‐beta	Before treatment	24	14 348	1.67 (95% CI: 1.12–2.50)		
	During treatment	23	11 428	2.01 (95% CI: 1.34–3.03)	1.20 (95% CI: 0.68–2.13)	0.53
Other disease‐modifying drugs	Before treatment	11	9872	1.11 (95% CI: 0.62–2.01)		
	During treatment	22	7055	3.12 (95% CI: 2.05–4.74)	2.80 (95% CI: 1.36–5.77)	0.0038

In our sensitivity analysis, we shortened the observation window to 1 year before and after treatment initiation, which did not affect our conclusions.

## 4 DISCUSSION

We followed 7174 patients starting interferon‐beta for MS and found no increased psoriasis risk compared to the period just before treatment. Thus, our study does not support the hypothesis that interferon‐beta triggers new onset of psoriasis. This result is consistent with studies investigating biological treatments targeting interferon pathways using anti‐interferon monoclonal antibodies, which were found to be ineffective in treating psoriasis.^12^, ^13^

Contrarily, in the control group treated with other DMDs, there was a rise in psoriasis cases during treatment. The distinct patterns observed in the control cohort make it even more unlikely that interferon‐beta would increase the risk of psoriasis.

A major strength of our study is the dataset covering all MS patients in Denmark, providing a solid foundation for analysis. To our knowledge, this is the first study to investigate the relationship between interferon treatment and the risk of psoriasis by comparing exposure data with control data. Previous knowledge comes exclusively from case reports.^2^

Our setup has not investigated whether interferon‐beta treatment can worsen existing psoriasis, as all patients with psoriasis prior to the study period have been excluded. Our method assumes that the risk of psoriasis is solely influenced by the medication. However, our study challenges this assumption due to the variable nature of psoriasis onset, which shows a bimodal age pattern, typically peaking in the 30s and 60s.^14^ However, the limited duration of our study period, restricted to 4 years, likely minimizes the impact of this divergence. Another important aspect may be the effect of frequent doctor visits in the period after DMD initiation, but we assume that such behavioral changes will be associated with a false‐positive finding.

The mechanisms underlying the development of psoriasis share similarities with those involved in MS, as both conditions are characterized by dysregulated immune responses.^15^ The connection is, however, poorly understood.^15^ Treatment with interferon‐beta may have a different influence on psoriasis pathogenesis in individuals with MS than in individuals without MS.

In summary, this registry‐based self‐controlled study does not support the notion that interferon‐beta acts as a trigger for psoriasis development.

## CONFLICT OF INTEREST STATEMENT

Melinda Magyari has served on the scientific advisory board, as a consultant for, and has received support for congress participation and speaker honoraria from Biogen, Sanofi, Roche, Novartis, Merck, Alexion, and Bristol Myers Squibb. The Danish MS Registry has received research support from Biogen, Genzyme, Roche, Merck, and Novartis. The other authors declare no competing interests.
